# *Psf2* plays important roles in normal eye development in *Xenopus laevis*

**Published:** 2008-05-19

**Authors:** Brian E. Walter, Kimberly J. Perry, Lisa Fukui, Erica L. Malloch, Jason Wever, Jonathan J. Henry

**Affiliations:** 1 Biology Department, Illinois Wesleyan University, Bloomington, IL; 2 Department of Cell and Developmental Biology, University of Illinois, Urbana, IL

## Abstract

**Purpose:**

Psf2 (partner of Sld5 2) represents a member of the GINS (go, ichi, ni, san) heterotetramer [1] and functions in DNA replication as a “sliding clamp.” Previous in situ hybridization analyses revealed that *Psf2* is expressed during embryonic development in a tissue-specific manner, including the optic cup (retina) and the lens [2]. This article provides an analysis of *Psf2* function during eye development in *Xenopus laevis*.

**Methods:**

A morpholino targeted to *Psf2* mRNA was designed to knockdown *Psf2* translation and was injected into specific embryonic cells during early cleavage stages in the frog, *Xenopus laevis*. Injected embryos were assayed for specific defects in morphology, cell proliferation, and apoptosis. Synthetic Psf2 RNA was also co-injected with the morpholino to rescue morpholino-mediated developmental defects. It is well known that reciprocal inductive interactions control the development of the optic cup and lens. Therefore, control- and morpholino-injected embryos were used for reciprocal transplantation experiments to distinguish the intrinsic role of *Psf2* in the development of the optic cup (retina) versus the lens.

**Results:**

Morpholino-mediated knockdown of *Psf2* expression resulted in dosage-dependent phenotypes, which included microphthalmia, incomplete closure of the ventral retinal fissure, and retinal and lens dysgenesis. Defects were also observed in other embryonic tissues that normally express *Psf2* including the pharyngeal arches and the otic vesicle, although other tissues that express *Psf2* were not found to be grossly defective. Eye defects could be rescued by co-injection of synthetic Psf2 RNA. Examination of cell proliferation via an antibody against phospho-histone H3 S10P revealed no significant differences in the retina and lens following *Psf2* knockdown. However, there was a significant increase in the level of apoptosis in retinal as well as forebrain tissues, as revealed by TUNEL (terminal deoxynucleotide transferase dUTP nick end labeling) assay.

**Conclusions:**

The results demonstrate intrinsic roles for *Psf2* in both retinal and to a lesser extent, lens tissues. Observed lens defects can mainly be attributed to deficiencies in retinal development and consequently the late phase of lens induction, which involves instructive cues from the optic cup. Developmental defects were not observed in all tissues that express *Psf2*, which could be related to differences in the translation of *Psf2* or redundant effects of related factors such as proliferating cell nuclear antigen (PCNA).

## Introduction

Integral to the process of DNA replication is the recruitment of protein complexes that function as “sliding clamps,” mediating the function of DNA polymerases alpha, delta, and epsilon during the initiation and elongation phases of replication [1-4]. One such sliding clamp is PCNA (proliferating cell nuclear antigen), which forms a ring-shaped trimeric complex and is well known for its roles in DNA replication [3,4]. However, PCNA has been shown to also be involved in other processes including cell cycle control and DNA repair [5,6]. Additional evidence suggests that PCNA may play roles in chromatin remodeling, RNA transcription, and tissue differentiation [4,6].

Another ring-like complex called GINS (go, ichi, ni, san) has recently been described in diverse organisms including *Saccharomyces cerivisiae* and *Xenopus laevis* [1,7]. Four evolutionarily conserved components, Sld5, Psf1 (i.e., partner of Sld5 1), Psf2, and Psf3, comprise the GINS heterotetramer. Studies have shown that GINS binds to the pre-RC in a cdc45-mediated and CDK-dependent manner, and the presence of GINS components is required for DNA synthesis to occur in *Xenopus laevis* egg extracts. The ring-like structure and specific interactions suggest that GINS, like PCNA, acts as a sliding clamp for DNA polymerase epsilon to promote initiation and continued elongation during DNA synthesis [1,8].

**Figure 1:**
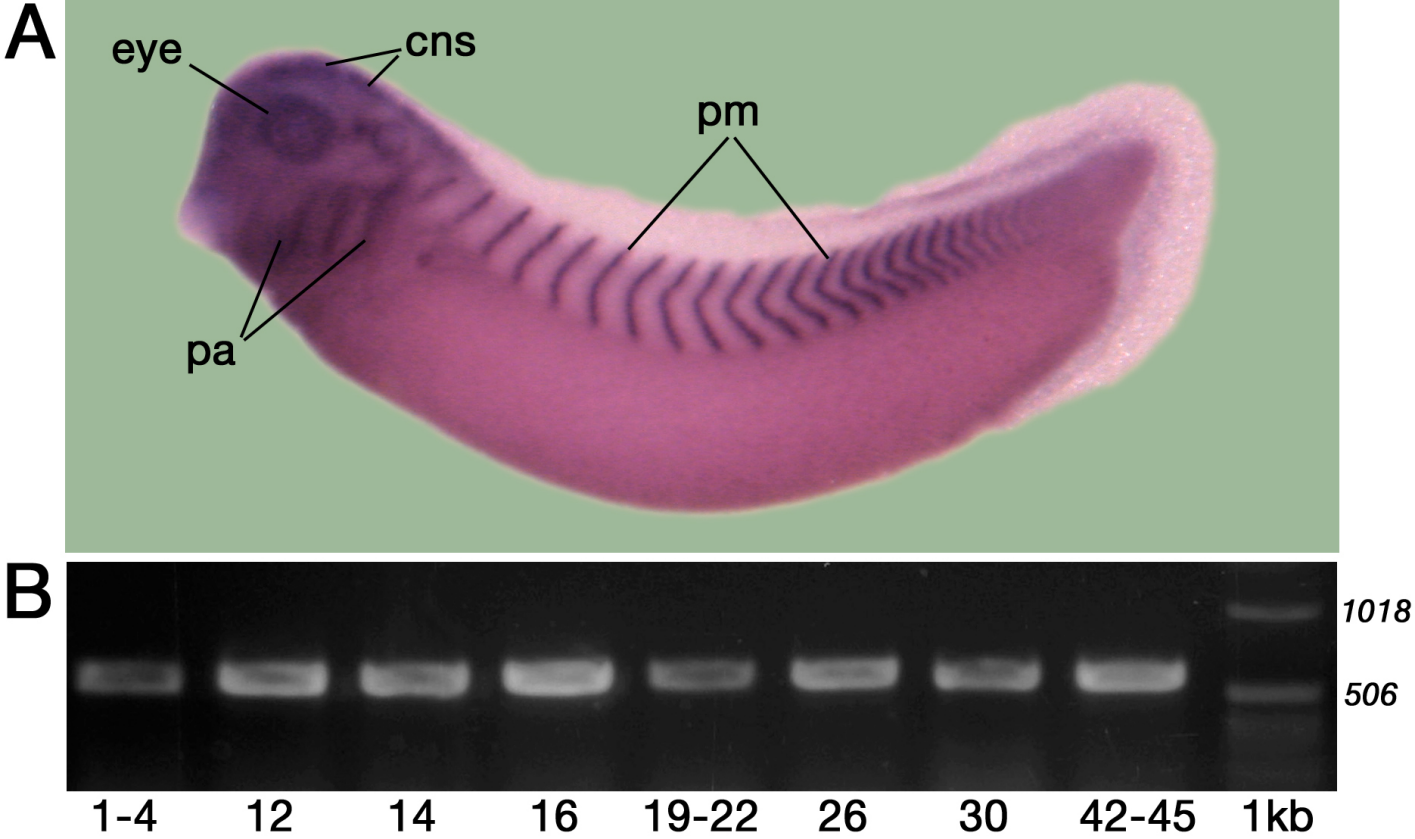
Embryonic expression of *Psf2*. **A**: An example of a whole mount in situ hybridization pattern showing localization of *Psf2* in specific embryonic tissues (stage 33) is shown. Note the expression in the brain (labeled as cns), the retina and lens of the eye (labeled as eye), mesoderm of the pharyngeal arches (labeled as pa), and in stripes representing a reiterated subset of the paraxial (somitic) mesoderm (labeled as pm). **B**: RT–PCR analysis of *Xenopus laevis Psf2* at different stages of embryogenesis, as noted (all stages follow those of [15]). A portion of 1 kb ladder was run for reference (labeled as 1kb). The 1018 bp and 506 bp bands are labeled. Expected *Psf2* PCR product is 577 bp. For simplicity, positive and negative control lanes are not shown here.

Recently, we found that *Psf2* (*partner of Sld5 2*) is significantly upregulated during the process of lens regeneration (a phenomenon also known as cornea-lens transdifferentiation) in the frog, *Xenopus laevis*, expressed sequence tag (EST) “H145” [2,9]. In situ hybridization during embryonic stages revealed a tissue-specific *Psf2* expression pattern including expression in both the developing retina and lens (Figure 1A and see the more thorough description in [2]). Interestingly, the spatial expression of *Psf2* differs from that of the other GINS components. Moreover, *Psf2* expression does not simply coincide with embryonic tissues that exhibit the highest rates of cell proliferation [2]. Likewise, these patterns of expression do not entirely match those of PCNA [2]. These observations indicate that various factors involved in DNA replication are deployed in different embryonic tissues and could have other functions. One report demonstrated that *Psf2* expression is activated in intrahepatic cholangiocarcinoma cells [10], and this gene also appears to play a role in chromosome segregation [11].

In this paper, we report studies examining the function of *Psf2* during development in the frog, *Xenopus laevis*, via morpholino**-**mediated knockdown of translation. Additionally, knockdowns were performed in conjunction with reciprocal tissue transplantation to ascertain the spatial-temporal action of Psf2 during eye development. Knockdown of *Psf2* in eye tissues led to a suite of developmental defects, demonstrating a role for *Psf2* in retinal, and to a lesser extent, lens development. Interestingly, analyses revealed no significant differences in cell proliferation within these eye tissues when compared to various controls. On the other hand, there was a significant increase in the level of apoptosis within the retina following morpholino knockdown of *Psf2*. Similar increases in apoptosis were not observed in all tissues known to express *Psf2,* such as the paraxial mesoderm. The data reveal tissue specific roles for *Psf2*, which extend beyond those related to DNA replication and cell proliferation.

## Methods

### Maintaining and handling *Xenopus laevis* adults and embryos

Adult *Xenopus laevis* were obtained from NASCO (Fort Atkinson, WI). Fertilized eggs were prepared following previously published protocols [12,13]. Embryos were reared in 1/20 normal amphibian media (1/20X NAM, [14]) at 16 °C unless otherwise indicated below. Embryos were staged according to reference [15].

### Reverse transcription polymerase chain reaction analyses

TRIzol reagent (Invitrogen, Carlsbad, CA) was used to extract total RNA from embryos at the following stages: 1–4, 12, 14, 16, 19–22, 26, 30, and 42–45. RNA was treated with RQ1 RNase-free DNase (Invitrogen, Carlsbad, CA) and purified using NucAway columns (Ambion, Austin, TX). Total RNA (5 μg) from each of the representative stages served as templates for reverse transcription reactions using oligo dT primers (Superscript III M-MLV RT; Invitrogen) according to the manufacturer’s instructions. cDNA was subsequently treated with RNase H (Invitrogen). The following set of primers (Invitrogen) was used for polymerase chain reaction (PCR): H145For: 5′-GTCATGGATGCCTCTGAGG-3′; H145Rev: 5′-GTGTGTTCTCAGCAGCCAGA-3′ (producing a product of 557 bp). PCR conditions for all reactions included 2.0 μl of the first strand cDNA (approximately 0.5 μg template DNA), 1.0 mM MgCl_2_, Platinum Taq polymerase, and reaction buffer (Invitrogen), 54 °C annealing temperature, and 30 cycles using a PTC-200 thermocycler (MJ Research, Waltham, MA). One nanogram of the H145 clone in pSPORT1 (Invitrogen) was used as a positive control.

### Morpholino oligonucleotide design

BLASTn analyses of various databases revealed numerous *Xenopus laevis Psf2* ESTs. None of the analyses exhibited any sequence variation in the targeted 5′ region. Morpholinos (MO) against *Psf2* called “Psf2MO” were synthesized by Genetools Inc. (Philomath, OR) to target the 5′ translational start site and adjacent 3′ open reading frame of the *Psf2* transcript. The targeted sequence of *Psf2* including the 5′ start site is as follows: 5′-GGATGCCTCTGAGGTCGAGTTCTTG-3′ (translation initiation site is underlined). Two *Psf2* specific morpholinos were used in this study. One morpholino recognized part of the translation initiation site and the downstream sequence (5′-CAAGAACTCGACCTCAGAGGCATCC-3′; complementary portion of start site is underlined). The second morpholino was identical in sequence but had a 3′ lissamine tag, which enabled lineage tracing. Both of these morpholinos provided identical results. In addition, a standard, untagged or lissamine-tagged random control morpholino (CONMO) was also used as a negative control (5′-CCTCTTACCTCAGTTACAATTTATA-3′) to assay for any non-specific effects of the injections or possible cytotoxicity associated with morpholinos. The latter sequence was designed against a human globin intron and is not known to target any *Xenopus laevis* sequences.

### Generation of RNAs

A cDNA was designed to generate full-length functional synthetic RNA, as an altered form of *Psf2* RNA (altPsf2 RNA), for injection into *Xenopus laevis* embryos. The sequence immediately downstream of the start site of *Psf2* was altered to prevent hybridization with Psf2MO while preserving the original protein coding sequence. This was accomplished via PCR to introduce third-base substitutions of each codon represented in the Psf2MO sequence. PCR conditions included: 100 ng template DNA, 1.0 mM MgCl_2_, Taq polymerase and reaction buffer (Invitrogen), 45 °C annealing temperature, and 30 cycles using a PTC-200 thermocycler (MJ Research). The following PCR primers were used to generate the altered cDNA, which was subsequently cloned into pCS2+ (Clonetech, Mountain View, CA) following digestion with ClaI and XbaI: upstream primer 5′**-**ACC*ATCGAT*ATGGAcGCtTCcGAaGTtGAaTTtTTaGC-3′ with underlined bases corresponding to the translation initiation site, bases in italics representing the ClaI restriction site, and lower case bases representing those altered from the original *Psf2* sequence; downstream primer: 5′-AGC*TCTAGA*GACAATTGCTTAGTAATCCTGTGACT-3′ with bases in italics representing the XbaI restriction site. The insert sequence was verified by the University of Illinois Biotechnology Center (Urbana, IL) using the ABI Prism Dye Terminator Cycle Sequencing “Ready Reaction” kit (ABI Prism, Foster City, CA). Rescue RNA was synthesized using the SP6 mMessage mMachine Kit (Ambion, Austin, TX) following PCR with SP6 and T3 primers.

RNA encoding green fluorescent protein (GFP) was also synthesized using a pCS2-GFP plasmid for use as a lineage tracer to follow the fate of the injected blastomeres for experiments using untagged morpholinos. GFP RNA was synthesized using the SP6 mMessage mMachine Kit (Ambion, Austin, TX) following digestion with NotI.

### Microinjection of embryos

**Figure 2:**
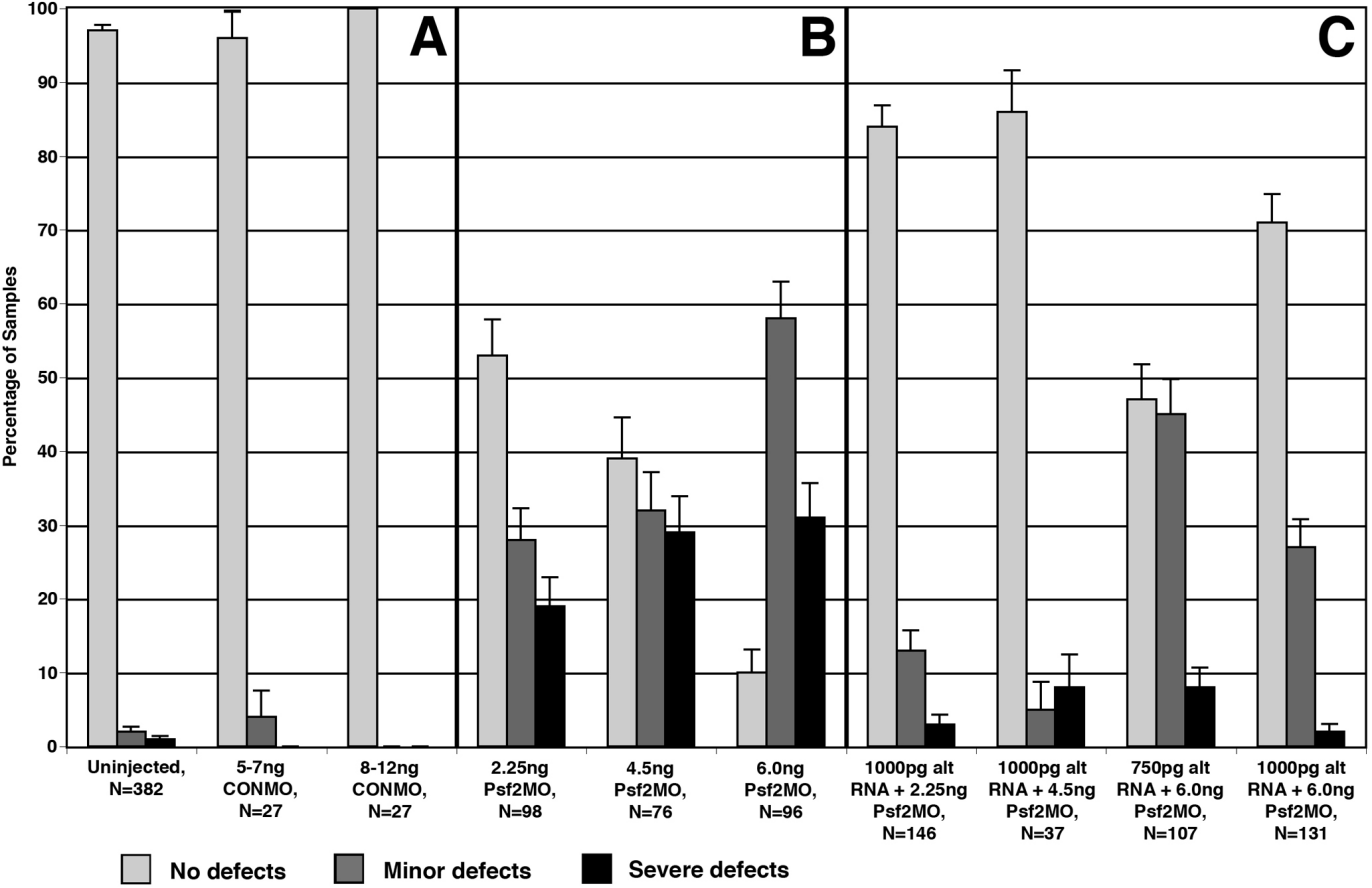
Summary of the effects of Psf2MO injection on eye development. Shading depicted in the key indicates categories of normal, minor, and severe eye defect phenotypes (see text for explicit definitions). **A**: Uninjected control embryos and those injected with the control morpholino (CONMO) exhibited minimal affects. **B**: Embryos injected with lissamine-tagged Psf2MO exhibit dose dependant eye defects. Note the increasing number and severity of eye defects with increasing doses of Psf2MO. **C**: Embryos co-injected with Psf2MO and altPsf2 RNA exhibit a dose dependent reduction in eye defects. Error bars indicate standard error.

For morpholino injections, embryos were dejellied and placed in 5% Ficoll in 1/20X NAM [14] for injection. The embryos were immobilized in clay-lined dishes for microinjection as previously described [16,17]. Following the manufacturer’s recommendations, the various morpholinos were dissolved at a stock concentration of 1 mM in ultra-pure distilled water (Sigma, St. Louis, MO) and stored at –80 °C. Thawed aliquots were heated to 65 °C for 10 min to ensure that they were completely dissolved, diluted to either 9 ng/nl or 18 ng/nl in distilled water, and injected at the required volumes (using a Harvard apparatus PLI100 Pico-Injector; Harvard Apparatus, Holliston, MA) to deliver specific quantities of the morpholino (quantities specified below; see also Figure 2).

To target a range of cell fates, specific quantities of morpholinos and RNAs were co-injected into single blastomeres at the two-cell, four-cell, and eight-cell stages. In some cases, multiple blastomeres within a single embryo were injected as noted. For the ease of comparison, all quantities are expressed as the equivalent to the morpholino quantities per individual blastomere at the eight-cell stage unless otherwise stated (see Figure 2). With the untagged Psf2MO morpholino, up to 125 pg of GFP mRNA was co-injected to serve as a lineage tracer. The embryos were cultured at 16 °C in daily changes of 1/20X NAM until at least stage 35–36 when the morphology of the eye is well developed. The various injections were repeated multiple times using different clutches of embryos.

### Reciprocal presumptive lens ectoderm (PLE) transplants

For transplant experiments, single cells were injected at the four-cell stage with 18 ng of Psf2MO (equivalent to 9 ng Psf2MO per blastomere at the eight-cell stage) to ensure a high level of defects. Up to 125 pg GFP RNA was co-injected as a lineage tracer. Embryos were then incubated in 1/20X NAM until stage 14 and transferred into 3/4X NAM for demembranation and surgery. After verifying the correct anterior expression of GFP in the injected embryos, the regions corresponding to the presumptive lens ectoderm were removed with glass microneedles and swapped with the corresponding regions of uninjected control embryos as described in reference [12]. On the day following surgery, embryos were equilibrated to 3/8X NAM and reared until unoperated sibling embryos reached stage 36.

### Histological analyses

Embryos were fixed in 4% paraformaldehyde in PBS, washed in PBS, and taken through a graded series of ethanol washes to 100% ethanol. The embryos were embedded in polyethylene glycol 400 distearate (Ruger, Irvington, NJ) and sectioned at a thickness of 12 µm as described in reference [18], or the embryos were transferred to xylene and embedded in Paraplast Plus (Fisher Scientific, Pittsburg, PA) and sectioned at a thickness of 8 µm [16]. Other specimens were fixed in MEMFA (3.7% formaldehyde, 100 mM MOPS, 2 mM EGTA, 1 mM MgSO_4_) for in situ hybridization [2] or TUNEL assays as described below. For immunological detection of lens proteins, primary cross-absorbed rabbit anti-lens antibody and goat anti-rabbit-rhodamine secondary antibody (Jackson ImmunoResearch Laboratories Inc., West Grove, PA) were used, as described in reference [18]. Other specimens were stained in Harris hematoxylin (Fisher Scientific, Hanover Park, IL) following published protocols [16,19]. Some lenses were measured using a filar micrometer, and lens diameters were recorded for each serial section to calculate the volume. These values were converted to a cylindrical volume based upon the thickness of the section, and the entire z-series was summed to obtain the total volume of each lens. Experimental lens volumes were then recorded as a percentage of either the contralateral control lenses, when appropriate, or an average of sibling control lens volumes. The percentages were then averaged for each experimental series.

### Analysis of cell proliferation via phospho-histone H3 S10P antibody labeling

As described above, fixed embryos were embedded in Paraplast Plus, sectioned at a thickness of 8 µm, and mounted on albumin-subbed slides [19]. A rabbit anti-phospho-histone H3 S10P antibody (histone H3 phosphorylated at serine 10, kindly provided by Dr. Craig Mizzen, University of Illinois-Urbana, Urbana, IL) was used to identify proliferating cells [20]. This antibody labels nuclei during prophase through the anaphase of mitosis. Slides were dewaxed in xylene, allowed to air dry, and rehydrated in PBT (PBS containing 0.1% Tween 20). Slides were then blocked in 5% dry milk in PBS for at least 2 h and incubated with rabbit anti-phospho-histone H3 S10P antibody (1:500) for at least 2 h. After washing six times in PBT, slides were then incubated with goat anti-rabbit fluorescein secondary antibody (1:100, Jackson ImmunoResearch Laboratories Inc.) in darkness for at least 1 h followed by six washes in PBT before mounting in 80% glycerol with 20% PBS and a 1:10,000 dilution of Hoechst 33342 to label all nuclei (Molecular Probes, Eugene, OR).

Due to the relatively low number of mitotic cells contained within the eye (e.g., nuclei labeled for phospho-histone H3 S10P) and the need to derive a significant set of measurements, labeled cells were counted in every serial section that contained eye tissues (i.e., the retina and lens) for each specimen examined. Five specimens injected with Psf2MO that exhibited the typical severe phenotype and five specimens injected with CONMO that exhibited the typical normal phenotype were examined. In each case, the control eyes (formed on the uninjected sides) and the contralateral eyes (formed on the morpholino-injected sides) were compared. Stage 36 embryos were selected because the differentiated eye phenotypes are readily apparent and *Psf2* is expressed in the retina and lens at that stage [2]. Lens and retinal areas were then determined for three random sections of each eye using the measure function in ImageJ by tracing the outlines of these structures. These areas were then used to determine the total volumes of the lens and retina by an ellipsoid volume formula derived by McKenney [21], modified to account for unequal spacing between the three sections used. An estimate of the total number of cells per unit volume of the retina and lens was then derived by counting all Hoechst 33342-labeled nuclei in three random sections containing lens tissue and six random sections containing retinal tissue for each specimen. These data were used to calculate the fraction of anti-H3 S10P positively stained cell nuclei in the retina and lens tissues for comparisons between control uninjected, Psf2MO injected, and CONMO injected cases. The standard deviation of the fraction of phospho-histone H3 S10P positive cells was calculated to evaluate the range and significance of each data set. The fractions of positively stained cells between Psf2MO cases and CONMO cases were compared using the Student's *t*-test. p values of less than 0.05 were considered significant.

### Analysis of cell death via TUNEL assay

The whole-mount TUNEL staining protocol was adapted from reference [22] with all washes and incubations performed with constant agitation. Morpholino-injected embryos were fixed in MEMFA as described above at stage 36 for no more than 1 h at room temperature then washed and stored in 100% methanol at –20 °C. Briefly, embryos were rehydrated in PBS, washed in PBT (0.2% Tween 20 in PBS), washed again in PBS, and washed in 1X terminal deoxynucleotidyl transferase (TdT) buffer for 30 min at room temperature. Recombinant TdT (Promega, Madison, WI) was added at a concentration of 150 U/ml with 0.5 µM digoxigenin dUTP (Roche, Indianapolis, IN), and embryos were incubated overnight at room temperature. The reaction was terminated in PBS with 1 mM EDTA at 65 °C and then washed with PBS at room temperature. The detection and chromogenic reactions were adapted from Harland [23]. Embryos were washed in PBT/BSA (PBS, 20 mg/ml BSA, 0.1% Triton-X; Sigma) and blocked in PBT/BSA with 20% goat serum for 1 h at room temperature. Embryos were further incubated overnight at 4 °C with anti-digoxigenin alkaline phosphatase antibody (Roche, Indianapolis, IN) that was diluted 1:2000 in PBT/BSA with 20% goat serum. Reactions were washed at least four times with PBT/BSA at room temperature and washed briefly with alkaline phosphatase (AP) buffer, then the stain was developed using nitro blue tetrazolium and 5-bromo-4-chloro-3-indolyl phosphate substrates. The reaction developed at room temperature for 30 min and was terminated by washing in AP buffer, and embryos were refixed in 4% paraformaldehyde for 1 h at room temperature to stabilize the staining pattern. Embryos were washed in 100% methanol and rehydrated in PBS. Using clear glass vials, embryos were bleached of their own natural pigmentation using 4% H_2_O_2_ for 1–2 h on a fluorescent light box, washed briefly in PBS, and then dehydrated with 100% methanol. Specimens were cleared in 2:1 benzyl benzoate/benzyl alcohol (BABB) for whole mount examination and then washed in three changes of 100% methanol and finally transferred to 100% xylene for embedding and sectioning as described above for histological analysis.

The number of TUNEL positive cells in these tissues was much greater, and therefore, estimates of the fractions of TUNEL positive cells in the retina and lens tissues were assessed by examining random sections through the eyes in control, uninjected embryos and morpholino-injected embryos. Control eyes formed on the uninjected sides and contralateral eyes formed on the MO-injected sides were examined in five specimens injected with Psf2MO (exhibiting the typical severe phenotype) and five specimens injected with CONMO (exhibiting the typical normal phenotype) at stage 36. For each embryo, three random sections from the uninjected side and three random sections from the morpholino-injected side were examined. Sections were selected that contained both retina and lens tissue. In each section, all TUNEL positive nuclei and all Hoechst 33342-labeled nuclei were counted separately for the lens and the retina. These data were used to obtain an estimate of the fraction of TUNEL positive cells in the retina and lens tissues for comparisons between the control, uninjected cases, the Psf2MO cases, and the CONMO injected cases. As above, the standard deviation of the fraction of TUNEL positive cells was calculated to determine the range and significance of each data set. Likewise, the fractions of positively stained cells between Psf2MO cases and CONMO cases were compared using the Student's *t*-test; p values of less than 0.05 were considered significant.

## Results

### *Psf2* expression: reverse transcription polymerase chain reaction analysis

The spatio-temporal expression pattern of *Psf2* has already been described for embryonic stages 14–37 as well as during the process of lens regeneration in *Xenopus laevis* (Figure 1A and [2]). Our earlier study indicated that Psf2 mRNA is detectable beginning at stage 14 and is present in discrete tissues at subsequent stages of development including the brain, retina, lens, ear, pharyngeal mesoderm, and paraxial mesoderm derived from the somites (Figure 1A). Here, we extended those analyses using RT–PCR to examine a broader range of stages. Though not quantitative, the results indicate that *Psf2* transcripts can be detected throughout all the stages examined (stages 1–45, Figure 1B).

### Knockdown of *Psf2* in developing embryos

Despite the fact that numerous orthologs have been cloned in a variety of organisms, few functional studies for *Psf2* have been undertaken. Careful functional analyses revealing a role for *Psf2* in DNA replication have only been performed in yeast [7] and in *Xenopus laevis* oocytes [1]. A *Drosophila* mutant is not available, but RNAi screens, which included the *Psf2* ortholog in *C. elegans*, revealed an embryonic lethal phenotype ([24], gene *F31C3.5* in wormbase). The differential expression pattern of *Psf2* during development and cornea-lens transdifferentiation in *Xenopus laevis* suggests that this gene may be necessary for the development of a specific subset of tissues including the retina and lens [25]. Therefore, morpholino knockdown experiments were performed to determine whether *Psf2* is necessary for normal development in *Xenopus laevis*.

Single blastomeres including those known to contribute descendents to the retina and lens were injected at the two-cell, four-cell, and eight-cell stages. These same cells also contribute descendents to the CNS, head and trunk neural crest, other placodes, mesoderm, and epidermis [26-28]. Embryos were injected unilaterally to allow for the contralateral, uninjected sides to serve as internal controls [29,30] and also to limit the potential for early embryonic lethality that could obscure the assessment of gene function. Expression of GFP and the lissamine fluorescent tracer were used to verify injections and the appropriate targeting of embryonic domains/tissues.

**Figure 3:**
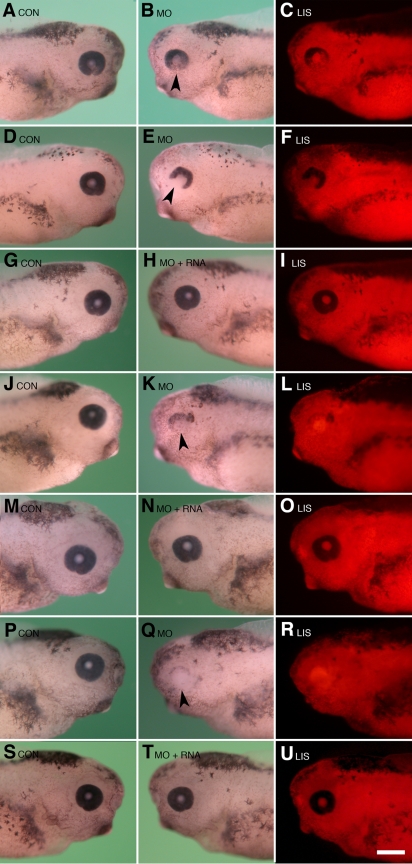
Results of *Psf2* morpholino knockdown and RNA rescue experiments. Dorsal is toward the top in each figure. **A**-**F**: Typical eye defects observed following unilateral injection of lissamine-tagged Psf2MO into single blastomeres at the two-cell stage (equivalent to 2.25 ng/cell at the eight-cell stage) are shown. **A**: Normal control (uninjected, CON) side is shown. This side is the normal part the embryo shown in **B**-**C**. **B**: Minor eye defect is observed on the Psf2MO-injected side (opposite that shown in **A**) as indicated by arrow. Note the slight decrease in retinal pigmentation in the ventral region and the reduced size of the optic cup compared to that shown in **A**. **C**: Corresponding fluorescence image to that shown in **B**, the image shows the distribution of the lissamine-tagged morpholino (LIS). **D**: Normal control, uninjected side is shown. This side is the normal part of the embryo shown in **E**-**F**. **E**: A severe eye defect phenotype is observed following Psf2MO injection. Note the smaller size of the eye and severe reduction in the ventral region of the optic cup, denoted by the arrow in **E**. **F**: The corresponding fluorescence image shows the distribution of the lissamine-tagged morpholino for the embryo shown in **E**. **G**-**I**: The typical result observed following the co-injection of 2.25 ng *Psf2* morpholino and 1000 pg synthetic altPsf2 RNA is shown. **G**: The normal control, uninjected side of the embryo shown in **H**-**I** is displayed. **H**: There was normal morphological development following the co-injection of Psf2MO (equivalent to 2.25 ng/blastomere at the eight-cell stage) and 1000 pg rescue RNA (altPsf2 RNA). **I**: The corresponding fluorescence image to that shown in **H** shows distribution of the lissamine-tagged morpholino. **J**-**L**: Typical severe eye defect is observed following the unilateral injection of lissamine-tagged Psf2MO into single blastomeres at the two-cell and four-cell stages (equivalent to 4.5 ng/blastomere at the eight-cell stage). **J**: The normal control, uninjected side of the embryo shown in **K**-**L** is pictured. **K**: Typical severe eye defect phenotype is observed following Psf2MO injection. Note the smaller size of the eye and severe reduction in the ventral region of the optic cup, denoted by the arrow in **K**. **L**: The corresponding fluorescence image shows the distribution of the lissamine-tagged morpholino for the embryo shown in **K**. **M**-**O**: The typical result is observed following the co-injection of 4.5 ng *Psf2* morpholino and 1000 pg synthetic altPsf2 RNA. **M**: The normal control, uninjected side of the embryo shown in **N**-**O** is displayed. **N**: Normal morphological development is observed following the co-injection of Psf2MO (equivalent to 4.5 ng/blastomere at the eight-cell stage) and 1000 pg rescue RNA (altPsf2 RNA). **O**: The corresponding fluorescence image to that shown in **N** shows the distribution of the lissamine-tagged morpholino. **P**-**R**: Typical severe eye defect is observed following unilateral injection of lissamine-tagged Psf2MO into single blastomeres at the two-cell and four-cell stages (equivalent to 6 ng/blastomere at the eight-cell stage). **P**: The normal control, uninjected side of the embryo shown in **Q** and **R** is shown. **Q**: The typical severe eye defect phenotype is displayed following Psf2MO injection. Note the severe reduction in the ventral region of the optic cup, denoted by the arrow in **Q**, and the overall lack of retinal pigmentation. **R**: The corresponding fluorescence image shows the distribution of the lissamine-tagged morpholino for the embryo shown in **Q**. **S**-**U**: The typical result is observed following co-injection of 6 ng *Psf2* morpholino and 1000 pg synthetic altPsf2 RNA. **S**: The normal control, uninjected side of the embryo shown in **T**-**U** is displayed. **T**: There is normal morphological development following the co-injection of Psf2MO (equivalent to 6 ng/blastomere at the eight-cell stage) and 1000 pg rescue RNA (altPsf2 RNA). **U**: The corresponding fluorescence image to that shown in **S** shows the distribution of the lissamine-tagged morpholino. The scale bar in **U** is equal to 450 µm.

The results of the various morpholino injection experiments performed are summarized in Figure 2. The typical phenotypes observed are shown in Figure 3. Eye morphologies were classified into three categories: (1) those with normal eyes, showing no morphological defects; (2) those with minor eye defects, exhibiting slightly diminished retinal pigmentation, smaller eye size (i.e., 50%–80% of the diameter of the contralateral control eye) or minimal ventral dysgenesis including phenotypes exhibiting incomplete closure of the ventral retinal fissure (Figure 3B); or (3) those with severe eye defects including cases with eyes less then 50% of the diameter of the contralateral control eyes, very little or no retinal pigmentation, extensive ventral eye dysgenesis (missing greater than 25% of the ventral-most edge of the retina), or no detectable eyes (Figure 3E,K,Q). Defects were almost always restricted to the progeny of the injected blastomeres (labeled by the fluorescent tracers).

**Figure 4:**
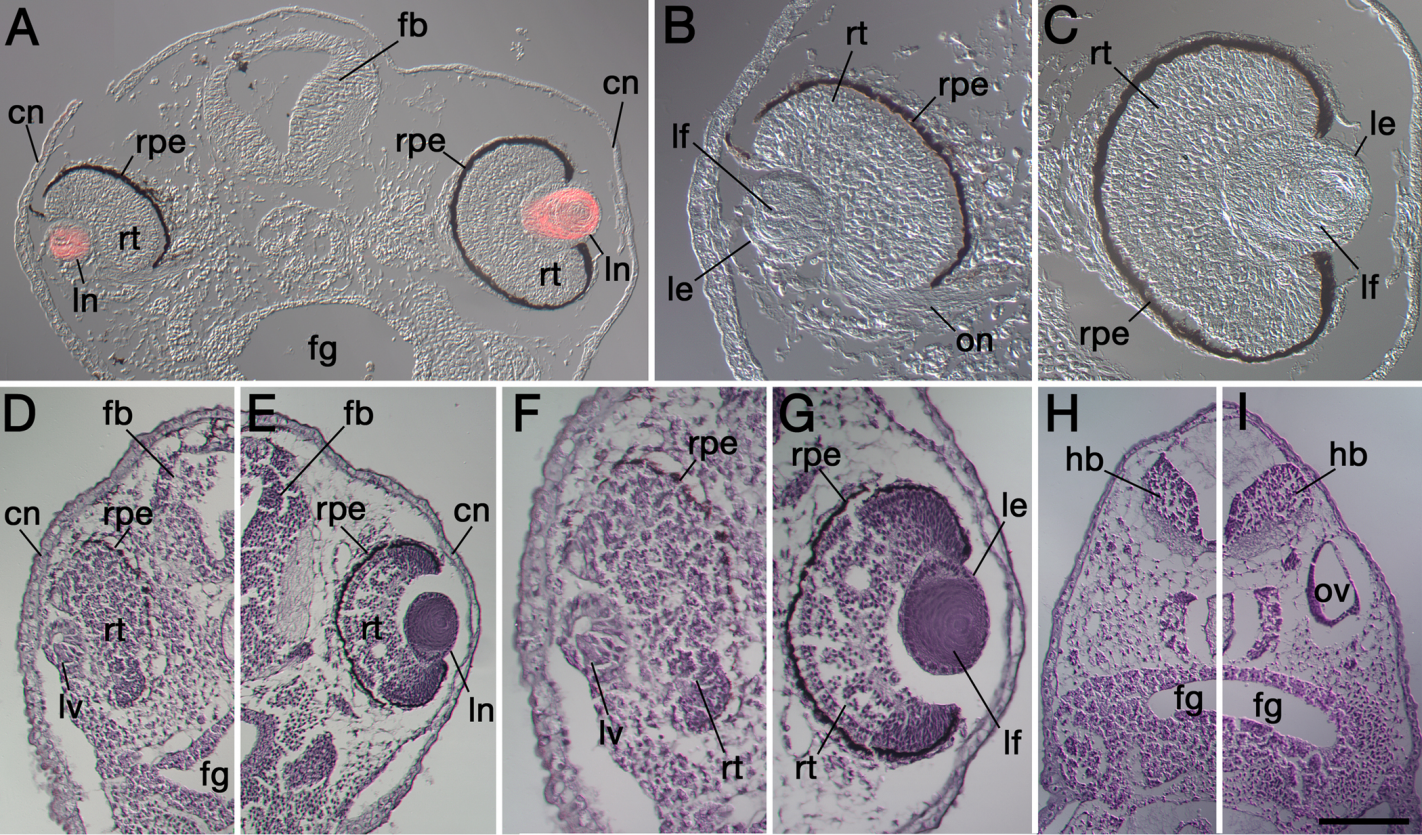
Transverse sections of specimens demonstrating severe eye defects following Psf2MO-mediated knockdown. Dorsal is toward the top in each figure. **A**: Image montage of a typical specimen shows the Psf2 morpholino-injected side on the left and the internal control, uninjected side on the right. This image was viewed with differential interference contrast (DIC). The red color in **A** shows overlain distribution of rhodamine fluorescence of secondary antibodies revealing immunoreactive lens crystallin proteins within both lenses. The cornea epithelium overlying the eye on the left is thicker compared to the uninjected side on the right, characteristic of undifferentiated embryonic ectoderm. Note that the lens and retina of the morpholino-affected eye are smaller and not as fully differentiated. The forebrain is also smaller and less differentiated on the left, Psf2MO-injected side. **B** and **C** are the higher magnification views of the left and right eyes shown in **A**, respectively. **D**-**E**: The left and right sides of the head of a second case stained with Hematoxylin/Eosin are shown. **F** and **G** are higher magnification views of the eyes shown in **D** and **E**, respectively. Note that the forebrain and retina are malformed on the left, Psf2MO-injected side shown in **D** and **F** compared to the control side shown in **E** and **G**. On the Psf2MO-injected side (**D**, **F**), only a small lens vesicle possessing a central lumen has formed. This lens vesicle exhibits some polarization and evidence of elongating primary fiber cells. Also note the retarded differentiation of the cornea in **D** and **F** compared to the respective control cornea shown in **E** and **G**. **H**-**I**: More posterior sections show the reduction in hindbrain size and the absence of the otic vesicle on the Psf2MO-injected side (**H**) compared to the normal pattern of development seen on the control, uninjected side (**I**). cn, cornea epithelium; fb, forebrain; fg, foregut; hb, hindbrain; le, lens epithelium; lf, lens fiber cells; ln, lens; lv, lens vesicle; on, optic nerve; ov, otic vesicle; rpe, retinal pigmented epithelium; rt, neural retina. The scale bar in **I** is equal to 160 µm in **A**-**B**, 85 µm in **B**-**C**, 170 µm in **D**-**E**, 100 µm in **F**-**G**, and 190 µm in **H**-**I**.

To determine if lenses were present, embryos were sectioned and the anti-lens antibody was used to detect lens proteins (following reference [18], Figure 4A). Lenses were present in all of the cases observed; however, they were smaller and generally proportional to the smaller retinas that formed (Figure 4A-G). The most severe cases lacked normal morphology, and the lens cells did not appear to be fully differentiated, were lacking overt fiber cells, and did not have a well established polarity. Ectopia lentis was observed in a few cases (data not shown). In comparison to control retinas, retinal tissues targeted with Psf2MO were smaller in size, lacked clearly organized neural retinal cell layers, and were without a fully developed retinal pigmented epithelium (Figure 4A-G).

Equivalent and higher doses of the control morpholino (CONMO) did not exhibit many noticeable defects other than a very low frequency of minor defects (i.e., slight reduction in eye size in some cases; Figure 2A). A very small, baseline level of eye defects was also seen in uninjected control embryos (Figure 2A). These results are consistent with those of contemporaneous studies in our laboratory showing that significant levels of eye defects are not observed with equivalent doses of the control morpholino [16,17]. These findings indicate that the significant levels of developmental defects detected in Psf2MO-injected cases cannot be simply attributed to the injection procedure or to non-specific cytotoxicity of the morpholinos.

When Psf2MO was localized to non-eye tissues, additional malformations were observed. For example, the overall size of the developing brain was reduced on the injected side (Figure 4A,D-E,H-I). Sections through these specimens revealed that the CNS on the morpholino-injected side contained fewer cells and even lacked a definitive proliferative (ventricular) zone (Figure 4A,D-E). The otic vesicles were reduced or absent on the Psf2MO side as well (Figure 4H-I). The incidence of eye defects was rare when Psf2MO was targeted to non-eye tissues, and this frequency was no higher than that observed in uninjected or CONMO-injected control embryos (data not shown). Some observations of particular toxicity effects for very high morpholino dosages have been reported in other studies [29,30] including axial truncations and aberrant cell death. Such defects were not observed for injections of control morpholinos with the doses used in this study (Figure 2A).

### Specificity of the Psf2MO knockdowns: RNA rescue experiments

To determine the specificity of Psf2MO knockdown, we initially obtained rabbit polyclonal antibodies raised against the Psf2 protein (as prepared by the Takisawa laboratory, Osaka University, Osaka, Japan [1]). Unfortunately, in our hands, this antibody failed to display a band of expected size in western blots using control, uninjected embryos (21,325 Da).

As an alternative approach, the specificity of the morpholino knockdown was verified by RNA rescue. Specifically, a cloned DNA variant was designed to generate an altered form of synthetic rescue RNA encoding full-length functional Psf2 protein (see Methods). RNA rescue represents a highly stringent test for specificity as the experiments rely on the functional capacity of the injected RNA. Cells were co-injected with both lissamine-tagged Psf2MO and the altered Psf2 rescue RNA (altPsf2 RNA) at different concentrations. The co-injection of altPsf2 RNA with Psf2MO significantly reduced the overall frequency and severity of eye defects in a dose dependent fashion (compare Figure 2B versus Figure 2C, and Figure 3P-R versus Figure 3S-U). Sibling control and Psf2MO-injected embryos were raised alongside these samples, and these confirmed that the overall effectiveness of the morpholinos injected during these experiments was as expected (Figure 2A-B).

### Reciprocal presumptive lens ectoderm transplants in Psf2MO-knockdown embryos

**Figure 5:**
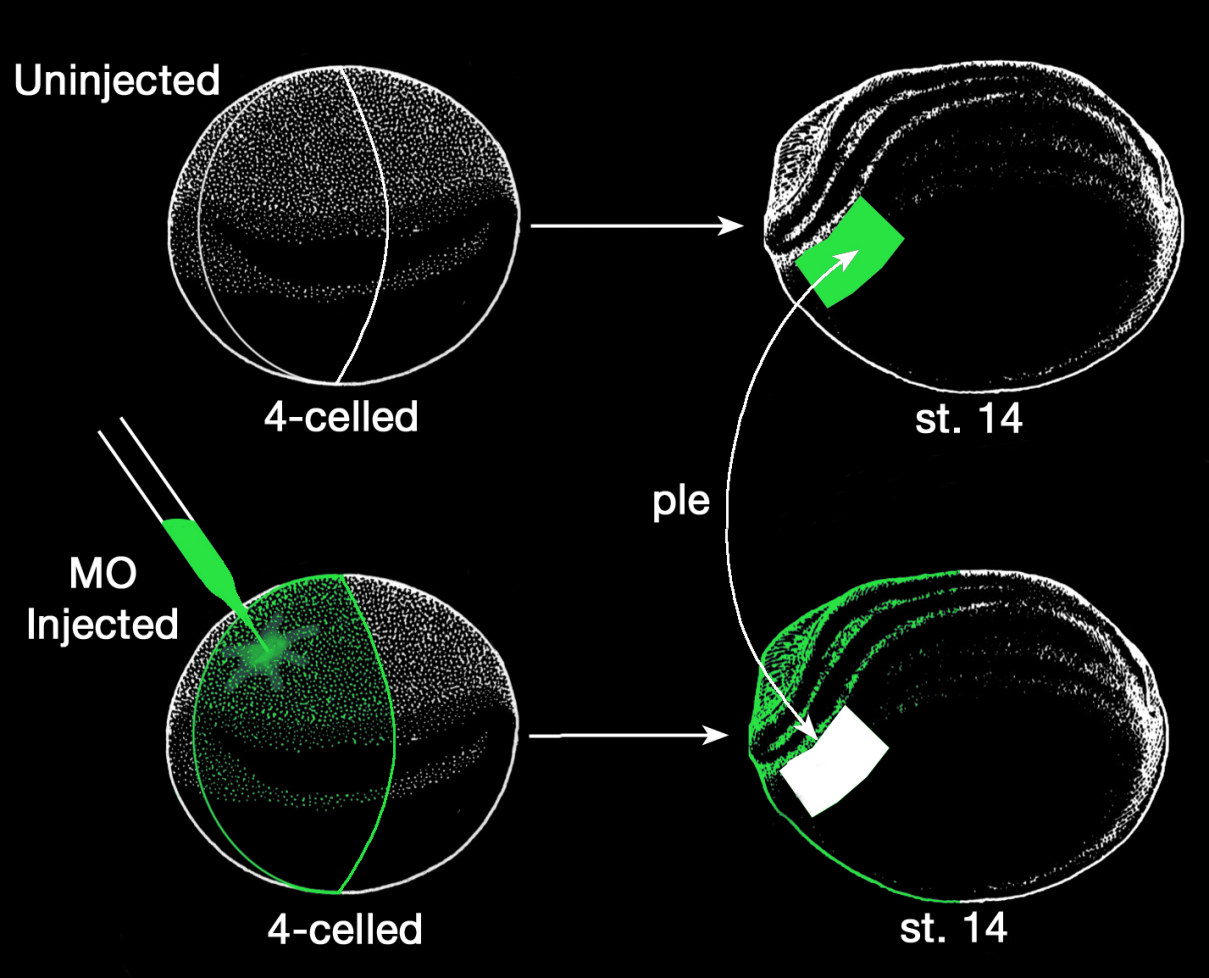
Diagrams illustrating the tissue transplantation experiment performed to localize *Psf2* function in the eye. This experiment involves reciprocal transplantation of stage 14 presumptive lens ectoderm (ple) between uninjected embryos (upper example) and morpholino (MO)-injected embryos (lower example). Single blastomeres were injected with morpholino at the four-cell stage as shown. Green color shows the distribution of the co-injected morpholino and GFP RNA tracer. See text for further details.

**Figure 6:**
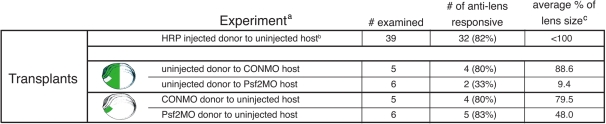
Summary of the data obtained from the reciprocal tissue transplantation experiments diagramed in Figure 5. See also the text and Figure 7. ^a^Green color denotes the presence of the morpholino. ^b^Data from Henry and Grainger is shown [18]. HRP stands for horseradish peroxidase. The exact lens sizes were not reported in that study but were less than the full size. ^c^See Methods for details on these measurements.

**Figure 7:**
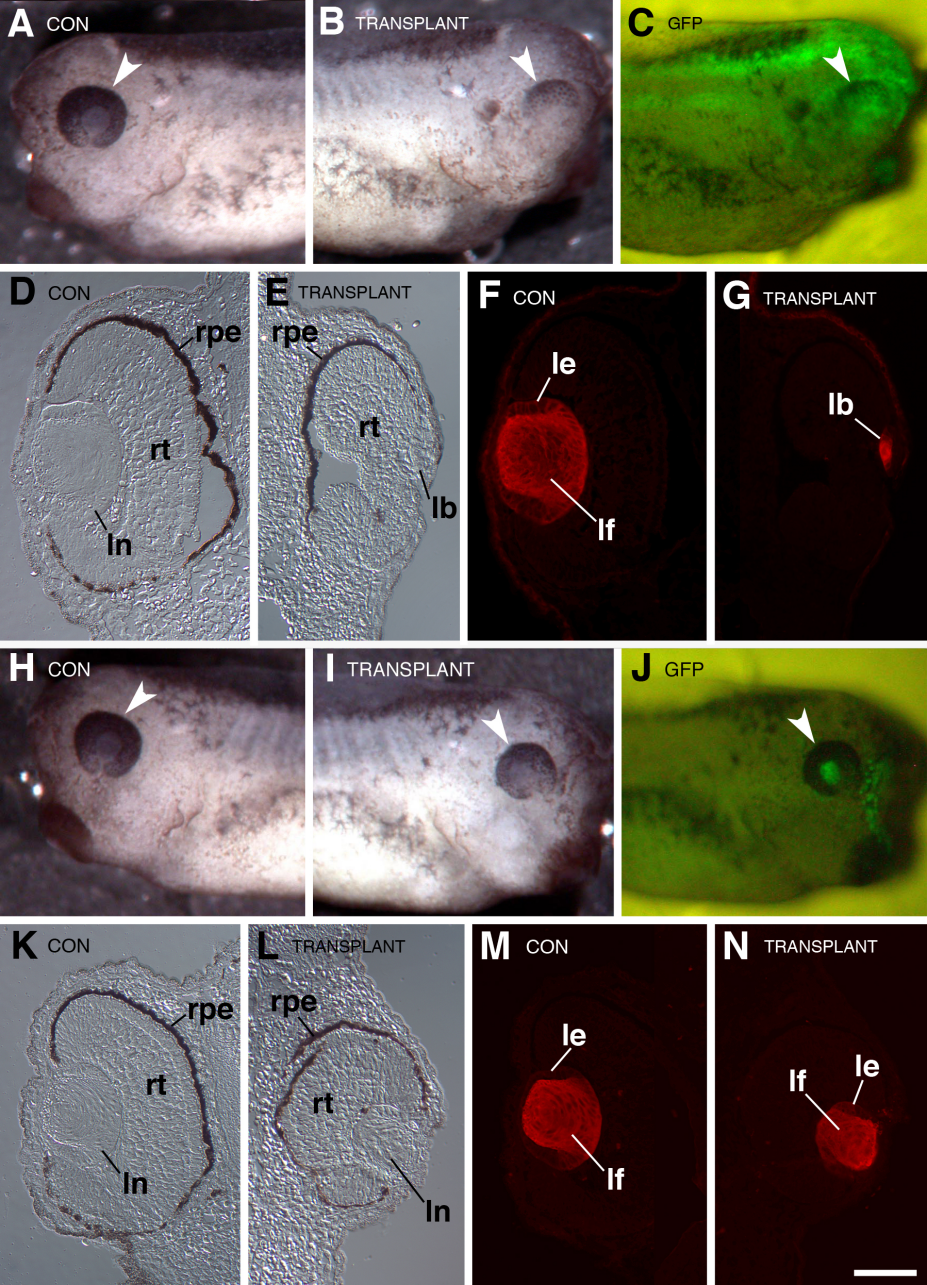
Examples of the results observed from reciprocal presumptive lens ectoderm transplants between control and Psf2MO-injected embryos. Dorsal is toward the top in each figure. Arrowheads point to eyes in the whole mounts shown in **A**-**C** and **H**-**J**. **A**-**G**: This example shows the typical result that is observed when the presumptive lens ectoderm from a control embryo is transplanted to the lens-forming region of a Psf2MO-injected host (see Figure 5, the text, and Figure 6). **A**: The view of the control (“CON”), unoperated side of the larva is shown. **B**: The view of the operated side that received the transplanted PLE (“TRANSPLANT”) shows abnormal development of the retina and lens. **C**: The whole mount fluorescence image corresponds to that shown in **B**, which reveals the location of the transplanted ectoderm via distribution of GFP expressed in the donor tissue (“GFP”). **D** and **E**: High magnification DIC images of transverse sections through the control side (**D**) and the operated side that received the transplanted tissue (**E**) are displayed. **F** and **G**: Corresponding immunofluorescence images show anti-lens antibody staining of the sections shown in **D** and **E**, respectively. Note formation of an abnormal retina and small lens body in **E** and **G**. **H**-**N**: This example shows the typical result that is observed when the presumptive lens ectoderm from a Psf2MO-injected embryo is transplanted to the lens-forming region of a control host (see Figure 5, the text, and Figure 6). **H**: The view of the control (“CON”), unoperated side of the larva is shown. **I**: The view of the operated side that received the transplanted PLE (“TRANSPLANT”) shows smaller overall size of the eye. **J**: The whole mount fluorescence image corresponds to that shown in **I**, which reveals the location of the transplanted ectoderm via distribution of GFP expressed in the donor tissue (“GFP”). **K**-**I**: High magnification DIC images of transverse sections through the unoperated, control side (**K**), and the side that received the PLE transplant derived from the Psf2MO-injected embryo (**L**), are shown. **M** and **N** display the corresponding immunofluorescence images showing anti-lens antibody staining of the sections presented in **K** and **L**, respectively. Note that the retina and lens formed on the operated side (**L** and **N**), although smaller compared to the unoperated side (**K** and **M**), exhibit fairly normal morphology. Labels are the same as those used in Figure 4. lb stands for lens body. The scale bar in **N** is equal to 450 µm in **A**-**C** and **H**-**J** and 80 µm in **D**-**G** and **K**-**N**.

It is well established that reciprocal inductive signals control the development of both the lens and the retina [31-36]. Because *Psf2* morpholino knockdowns perturb the development of both of these structures, it is important to distinguish between primary intrinsic requirements of *Psf2* function in the lens and/or retina and possible secondary effects related to defects in their reciprocal induction. To examine this, we combined morpholino injections with reciprocal transplantation of the presumptive lens ectoderm (Figure 5). Embryos were co-injected at the four-cell stage to produce severe eye defects (e.g., phenotypes seen in Figure 3K,Q). Injected embryos were raised to stage 14, and those with proper GFP expression in the presumptive retina and lens were used for reciprocal presumptive lens ectoderm transplants with uninjected control embryos (see Methods and Figure 5). Specimens were then raised to stage 36 and scored for eye and lens defects. Larvae with morpholino-containing donor tissue were scored for the presence of GFP in tissues derived from the donor transplant (i.e., the lens and head ectoderm), and morpholino-containing hosts were examined for GFP in the retina. Those that met the above conditions with transplants correctly located over the eye were sectioned and subsequently analyzed for the presence of lens proteins using the anti-lens antibodies. Results are presented in Figure 6 and Figure 7A-N. A set of control experiments was also performed using the control morpholino (CONMO) to assess the background rate of eye defects associated with the surgeries (Figure 6). Both control and morpholino-injected embryos not used in the transplant experiments were raised alongside the transplant embryos, and these confirmed that the overall effectiveness of the morpholinos injected during these experiments was as expected.

The CONMO host specimens containing uninjected transplants produced antibody positive lenses with an 80% frequency, and the lenses produced were on average 88.6% the volume of the contralateral, unperturbed lenses (Figure 6). Similar results were observed with CONMO donors and uninjected hosts in which antibody positive lenses formed 80% of the time and the lenses were 79.5% the size of the control lenses. In both cases, the lenses that formed from the donor tissue were well differentiated. These results are similar to those reported by Henry and Grainger [12] in which stage 14 control presumptive lens ectoderm was transplanted to the presumptive lens ectoderm region of another stage 14 control embryo. In those cases, antibody positive lenses were formed in 82% of the cases (Figure 6 and [12]).

Specimens with Psf2MO-targeted retinas and control donor presumptive lens ectoderm developed malformed retinas with morphologies consistent with those observed in Psf2MO-injected embryos. Lentoid tissues with positive responses to the anti-lens antibody were produced in only 33% of the cases (Figure 6, Figure 7A-G). Those tissues that were responsive to the antibody were very small (at 9.4% volume compared to the contralateral lenses, Figure 7D-G), resembling small lens vesicles or thickened lens placodes. Furthermore, none of these tissues contained obviously differentiated fiber cells indicative of normal lenses.

Conversely, Psf2MO-containing donor presumptive lens ectoderm transplanted to the presumptive lens region of uninjected control hosts produced lenses 83% of the time (Figure 6 and Figure 7H-N). This frequency of lens formation from these transplants is similar to the frequencies described above for the various control transplantation experiments. However, lenses that formed averaged only 48.0% of the volume of the contralateral lenses overall but generally exhibited normal lens polarity including differentiated primary and secondary fiber cells and lens epithelial tissue (Figure 7K-N).

### Examination of cell proliferation and apoptosis

**Figure 8:**
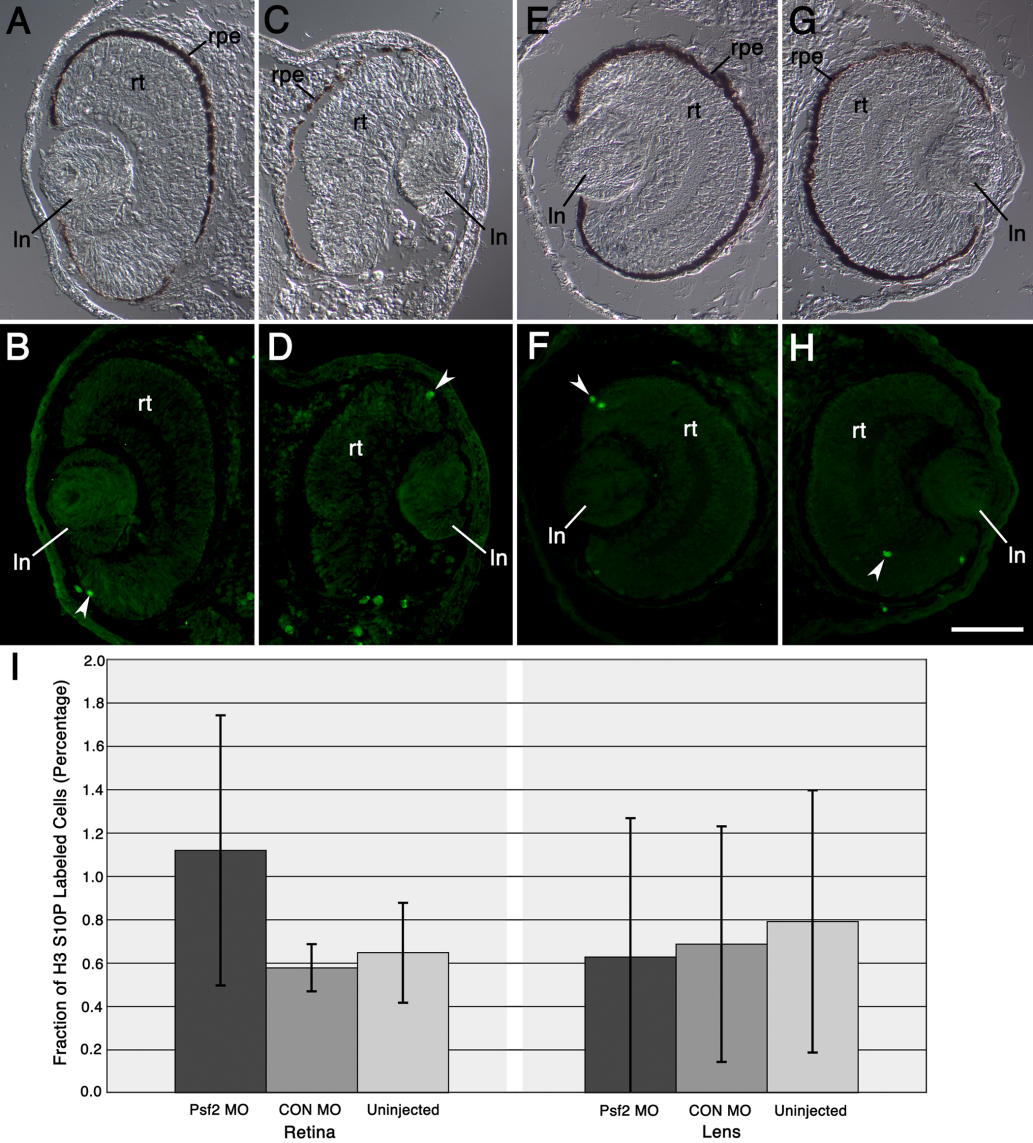
Effects of Psf2MO and CONMO injections on cell proliferation in the neural retina and lens. **A**-**H**: Transverse sections of eyes in Psf2MO-injected and CONMO-injected embryos show corresponding pairs of differential interference contrast and fluorescence micrographs. Fluorescence micrographs in **B**, **D**, **F**, and **H** show distribution of proliferating cells labeled with anti-phospho-histone H3 S10P antibody (green). White arrowheads point to examples of these labeled cells within the retina. **A** and **B**: The normal, control eye derived from the uninjected side of one example is displayed. **C** and **D**: Opposite, defective eye derived from the Psf2MO-injected side of the same embryo shown in **A**-**B** is displayed in these panels. **E** and **F**: The normal, control eye derived from the uninjected side of another embryo is shown. **G** and **H**: Opposite, normal-appearing eye derived from the CONMO-injected side of the same embryo shown in **E**-**F** is displayed in these panels. **I**: Graphical depiction of the levels of cell proliferation in the neural retina and the lens is shown. Bars represent the mean fraction of histone H3 S10P labeled cells (depicted as a percentage along the y-axis) while the different tissues and conditions examined are depicted along the x-axis, as indicated. Error bars representing the standard deviation are also shown. See Methods for further details explaining the preparation of this data. Labels are the same as those used in Figure 4. Scale bar in **H** equals 100 µm.

**Figure 9:**
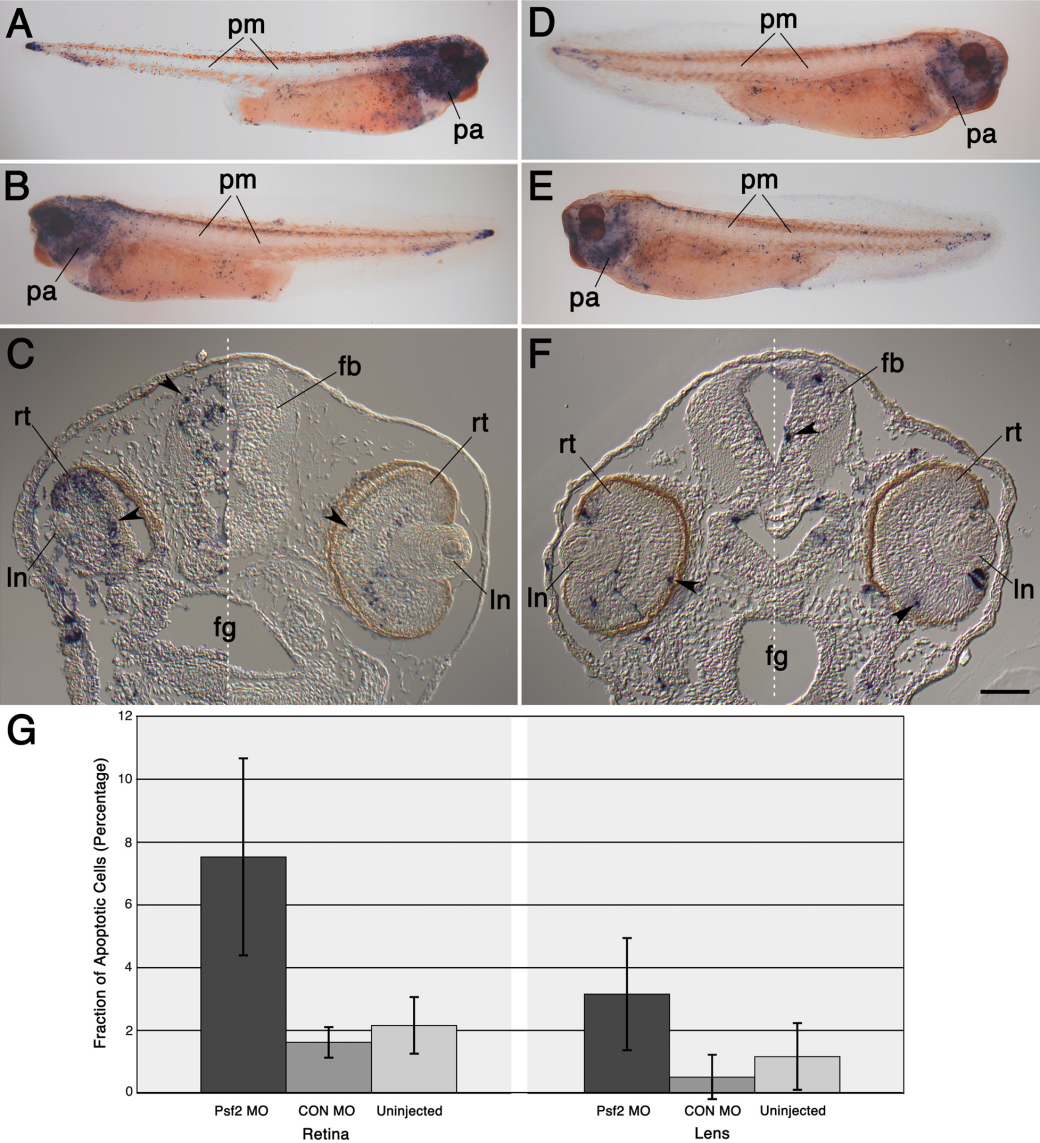
Effects of Psf2MO and CONMO injections on the level of apoptosis. **A-B,D-E**: Whole mount examples show gross distribution of apoptotic cells (containing blue colored NBT-BCIP precipitate). These whole mount embryos have been cleared with BABB. **A** and **B**: These views of an embryo show sides derived from Psf2MO-injected and uninjected blastomeres, respectively. **C**: Transverse section through the head of a Psf2MO-injected embryo is shown. The white dashed line separates the side containing tissues derived from the Psf2MO-injected blastomere (on the left side of the figure) from those derived from the uninjected blastomere (on the right side of the figure). **D** and **E**: These views of an embryo show sides derived from CONMO-injected and uninjected blastomeres, respectively. **F**: Transverse section through the head of a CONMO-injected embryo is shown. The white dashed line separates the side containing tissues derived from the CONMO-injected blastomere (on the left side of the figure) from those derived from the uninjected blastomere (on the right side of the figure). Note the increased level of apoptosis in head tissues derived from Psf2MO-injected cells, especially in the forebrain and neural retina (e.g., compare **A** versus **D** and **C** versus **F**). Black arrowheads point to examples of labeled apoptotic cells within the retina and brain. **G**: A graphical depiction of the levels of apoptosis in the neural retina and the lens is displayed. Bars represent the mean fraction of apoptotic cells (depicted as a percentage along the y-axis) while the different tissues and conditions examined are depicted along the x-axis, as indicated. Error bars representing the standard deviation are also shown. See Methods for further details explaining the preparation of this data. Labels are the same as those used in Figure 1 and Figure 4. Scale bar in **F** equals 600 µm for **A**-**B** and **D**-**E** and 110 µm for **C** and **F**.

Specimens were examined for differences in the levels of cell proliferation and cell death specifically in the retina and the lens following injections with Psf2MO and CONMO (Figure 8 and Figure 9). No significant differences were detected in the levels of proliferation in lens or retinal tissues compared to the controls (Figure 8). Within the retina, labeled cells were mainly located within or in close proximity to the region of the ciliary margin. Within the lens, these were situated within the lens epithelium. The presence of Psf2MO did not significantly influence the fraction of mitotic cells in the lens or retina (p=0.12 and 0.88, respectively, relative to CONMO injection). If anything, there was a slight increase in the fraction of mitotic cells in the retina following injections of Psf2MO (Figure 8I). On the other hand, a threefold to fourfold difference was detected in the level of apoptosis in the retina in the Psf2MO-injected embryos (Figure 9). In the retina, the fraction of apoptotic cells was significantly higher in the Psf2MO cases than in the CONMO cases (p=0.013). Similarly, significant increases in the level of apoptosis were also measured in the forebrain following Psf2 morpholino knockdown (Psf2MO mean=7.3%, SD=2.9%; CONMO mean=2.1%; SD=0.6%; uninjected control mean=1.7%, SD=1.1%). The fraction of apoptotic cells was significantly higher in the Psf2MO-injected cases compared to the CONMO-injected cases (p=0.011). Apoptotic cells were seen throughout the retina but predominantly located within the differentiating neuronal layers. A slight increase in the level of apoptosis was also seen in the lens (Figure 9G) where the fraction of apoptotic cells in the lens differed significantly between Psf2MO-injected and CONMO-injected cases (p=0.03) but not when compared to the uninjected controls (p=0.07). Increases in the level of apoptosis were also noticed in the pharyngeal region and the gut (Compare Figure 9A versus Figure 9D and Figure 9C versus Figure 9F). However, not all tissues that express *Psf2* exhibited these increases. For instance, there was no noticeable difference in the level of apoptosis in the somitic mesoderm in the trunk and tail following a pattern that mirrors the distinct repeated (striped) Psf2 mRNA expression pattern seen in this tissue (for instance, compare the whole mount expression pattern shown in Figure 1A, and those more specifically described by Walter and Henry [2], to the TUNEL staining patterns shown in Figure 9A-B,D-E).

## Discussion

### Morpholino-mediated knockdown of *Psf2* leads to specific eye defects

Morpholinos have been shown to be reliable for the analysis of gene function in *Xenopus laevis* [30,37,38]. They are stable and effective quite late in development (into the early tadpole stages in *Xenopus laevis* [29]). The morpholino knockdown studies reported here clearly demonstrate an important role of *Psf2* in the development of the eye. Other defects were also observed in brain tissues where *Psf2* is also normally expressed. When *Psf2* morpholino was targeted to non-eye tissues, defects were not observed in the eye above the low levels of background minor defects seen in uninjected or CONMO-injected embryos. These results demonstrate that the defects seen in the eye are directly correlated to the presence of Psf2MO in those progenitors. Furthermore, co-injection of rescue altPsf2 RNA with Psf2MO significantly reduced both the frequency as well as the severity of the defects observed versus those seen using comparable morpholino dosages without altPsf2 RNA (compare Figure 2B,C). These observations corroborate the specificity of the *Psf2* morpholino knockdown effects in this study.

In situ hybridization analysis shows that *Psf2* is expressed in the anterior CNS of neurula stage embryos particularly in the region of the forebrain and presumptive retina [25]. During the later stages of optic cup development, *Psf2* expression continues in the lens and optic cup and more intensely in the ciliary marginal zone [25]. The latter has been shown to be the site of proliferative activity responsible for increased growth of the retina [39]. Other tissues also express *Psf2* including the pharyngeal mesoderm and paraxial mesoderm within the tail (Figure 1A and see [2]). Interestingly, there were no significant differences noted in the level of proliferation in the retina or the lens following injections with Psf2MO. On the other hand, there were elevated levels of apoptosis particularly in the retina. Apoptotic cells were mainly seen in the differentiating layers of the retina. This may imply that *Psf2* is required at some level for the differentiation or survival of retinal cells. Although elevated levels of apoptosis were also detected in other tissues that express *Psf2* (including the forebrain and the pharyngeal region) following Psf2MO injections, we did not observe similar increases in the paraxial mesoderm of the tail. These findings suggest that *Psf2* may be important for only a subset of tissues that express this gene. These observations could also be related to differences in the translation of the protein or to redundant effects of other related factors such as PCNA.

### The role of *Psf2* in optic cup development and lens induction

Lens formation is normally directed by two phases of induction including an early phase that occurs during gastrulation (stages 10.5–19) and a late phase that occurs when the developing optic vesicle contacts the presumptive lens ectoderm (stages 19+; see [12,18]). Furthermore, the presumptive lens ectoderm also provides reciprocal signals for proper morphogenesis of the optic cup [31-36]. Therefore, it is important to distinguish between intrinsic and extrinsic roles of particular genes in the development of retinal and lens tissues. Reciprocal transplants using control, uninjected tissues and those affected by *Psf2* morpholino knockdown demonstrate a key role of *Psf2* in retinal development and a secondary, indirect function for *Psf2* in lens induction.

The results of the reciprocal transplant experiments indicate that the presence of CONMO in the presumptive lens ectoderm (PLE) does not adversely affect lens development beyond that normally attributed to this type of surgery. Only slight reductions in both the frequency of lens formation and the volume of the lens were observed. In fact, the results of these control experiments are comparable to those reported by Henry and Grainger [12,18] for transplantation experiments involving non-morpholino-injected embryos (Figure 6).

On the other hand, the results of experiments using Psf2MO are quite different and demonstrate an intrinsic role for *Psf2* in the development of the retina. In the transplants involving Psf2MO-injected retinal tissue, the retinas exhibited dysmorphogenesis similar to those observed in the initial injection morpholino experiments using comparable doses. In contrast, the transplants involving normal retinal hosts and *Psf2*-injected donor presumptive lens epithelium generally resulted in the hosts forming well differentiated retinas. A slight decrease in eye size overall can be attributed to the transplantation surgery as discussed above. Considering that the retinas are not malformed or degenerated, knockdown of *Psf2* in the lens does not appear to appreciably disrupt the ability of the lens to produce inductive signals or to direct retinal development.

*Psf2* was also found to have an intrinsic role in the development of the lens, albeit to a lesser extent than within the retina. *Psf2* morpholino-containing presumptive lens ectoderm (stage 14), transplanted to the lens forming a region of equivalent-stage uninjected hosts, formed properly-differentiated lenses at a frequency consistent with both CONMO injected and uninjected control transplants (see Figure 6 and [18]). This demonstrates that the presumptive lens ectoderm, even with diminished Psf2 function, is still able to receive and respond to the inductive signals from an unaffected optic cup (Figure 7L,N). These lenses were, however smaller in size compared to the control, contralateral lenses (Figure 6 and Figure 7K-M). The difference in lens size between the Psf2MO lenses (average of 48.0% of normal size) and the controls (e.g., using the control morpholino resulting in lenses average of 79.5% of normal size; Figure 6) indicates that *Psf2* may play an intrinsic role for full development of the lens. These data coincide with our observations that the levels of apoptosis increased slightly in lenses derived from Psf2MO-injected cells. (Figure 9G).

Our experiments demonstrate that lenses fail to form normally when the retina is perturbed via *Psf2* knockdown. Unlike the transplants involving Psf2MO donors, transplants of uninjected stage 14 presumptive lens ectoderm to stage 14 hosts that were injected with Psf2MO resulted in a marked reduction in both lens formation frequency and lens quality (Figure 6 and see Figure 7E,G). Henry and Grainger [18] showed that when any further inductive signaling to stage 14 presumptive lens ectoderm is curtailed, the ectoderm is unable to consistently form lens tissue. None of the cases formed lens tissue when the presumptive lens ectoderm was explanted, and only 15% of the cases formed lens tissues when the anterior neural plate tissue that included the retinal rudiments was removed. When *Psf2* function is perturbed in the optic cup, proper retinal development is inhibited. Consequently, the optic vesicle is unable to produce key inductive signals required for normal lens development (i.e., the late phase of lens induction is compromised).

### *Psf2* expression is not directly correlated with embryonic patterns of cell proliferation

Recent analyses have examined the expression of a variety of cell cycle regulatory and DNA replication genes (e.g., *cdk2*, *cyclin E*, *cdc45*, *PCNA*, *Sld5*, *Psf1*, *Psf2*, and *Psf3*) [2,40,41]. Although many of these genes are expressed together in neural tissues, each one exhibits unique spatial domains within other tissues. For instance, *Psf2* is expressed only in a subset of tissues including the somitic mesoderm, the pharyngeal arches, the otic vesicles, the anterior CNS, and the eye [2]. These findings are somewhat unexpected as genes important for DNA replication, cell cycle control, and cell division are generally thought to be expressed ubiquitously within developing organisms.

Proliferation patterns have been determined in *Xenopus laevis* embryos using BrdU incorporation to monitor DNA replication [42] and anti-phospho-histone H3 S10P antibodies to detect mitotic cells [43]. These studies reveal high levels of proliferation in the developing central nervous system and lesser degrees of cell division in the epidermal ectoderm. As mentioned above, the expression patterns of various DNA replication factors are spatially restricted and do not necessarily correlate with all sites of proliferation within the developing embryo. This suggests that several of these genes including *Psf2* may have roles beyond that of DNA replication and cell proliferation.

The activity of GINS (comprised of *Psf1*, *Psf2*, *Psf3,* and *Sld5*) appears to be analogous to that of PCNA, a trimeric sliding clamp, which also binds to a variety of DNA polymerases. Although PCNA is involved in DNA replication, several studies suggest that it may indeed function in different capacities. These roles not only include aspects of cell cycle control and DNA repair [3] but may also include roles in differentiation and transcription [44-46]. Evidence such as this emphasizes the delicate balance between cell proliferation and fate determination that is required for proper development. Studies in which a cell’s ability to exit the cell cycle was perturbed have demonstrated shifts in cell fate from those normally anticipated [47,48]. Perturbing *Psf2* activity in the retina may likewise alter the cells’ usual exit from proliferation, forcing them to either remain in an undifferentiated state, proceed along an incorrect determination pathway, or undergo apoptosis, depending on the extrinsic and intrinsic factors available at that stage of development. This may therefore disrupt the inductive potential of the retina and subsequently alter the development of the lens.
